# Chronic kidney disease in children: the global perspective

**DOI:** 10.1007/s00467-006-0410-1

**Published:** 2007-02-20

**Authors:** Bradley A. Warady, Vimal Chadha

**Affiliations:** 1 Department of Pediatrics, Section of Nephrology, The Childrens Mercy Hospital, Kansas City, MO USA; 2 Department of Pediatrics, Section of Nephrology, Virginia Commonwealth University Medical Center, Richmond, VA USA; 3 University of Missouri–Kansas City School of Medicine, The Childrens Mercy Hospital, 2401 Gillham Road, Kansas City, MO 64108 USA

**Keywords:** Chronic kidney disease, End-stage renal disease, Children, Epidemiology, Renal replacement therapy

## Abstract

In contrast to the increasing availability of information pertaining to the care of children with chronic kidney disease (CKD) from large-scale observational and interventional studies, epidemiological information on the incidence and prevalence of pediatric CKD is currently limited, imprecise, and flawed by methodological differences between the various data sources. There are distinct geographic differences in the reported causes of CKD in children, in part due to environmental, racial, genetic, and cultural (consanguinity) differences. However, a substantial percentage of children develop CKD early in life, with congenital renal disorders such as obstructive uropathy and aplasia/hypoplasia/dysplasia being responsible for almost one half of all cases. The most favored end-stage renal disease (ESRD) treatment modality in children is renal transplantation, but a lack of health care resources and high patient mortality in the developing world limits the global provision of renal replacement therapy (RRT) and influences patient prevalence. Additional efforts to define the epidemiology of pediatric CKD worldwide are necessary if a better understanding of the full extent of the problem, areas for study, and the potential impact of intervention is desired.

## Introduction

Most epidemiological information on chronic kidney disease (CKD) originates from data available on end-stage renal disease (ESRD), the terminal stage of CKD when treatment with renal replacement therapy (dialysis or transplant) becomes necessary to sustain life. Little information is available on the prevalence of earlier stages of CKD, as patients are often asymptomatic. The epidemiological studies that have been performed provide evidence that ESRD represents the “tip of the iceberg” of CKD and suggest that patients with earlier stages of disease are likely to exceed those reaching ESRD by as much as 50 times [1]. Worldwide, the number of patients with CKD is rising markedly, especially in adults, and CKD is now being recognized as a major public health problem that is threatening to reach epidemic proportions over the next decade [2]. In North America, up to 11% of the population (19 million) may have CKD [1], and surveys in Australia, Europe, and Japan describe the prevalence of CKD to be 6–16% of their respective populations [3, 4]. In North America alone, more than 100,000 individuals entered ESRD programs in 2003 (adjusted incidence rate: 341 new cases per million population), with a prevalence count of more than 450,000 as of December 2003 (prevalence rate: 1,509 per million population) [5]. Not surprisingly, the cost of treating patients with ESRD is substantial and poses a great financial challenge. The economic cost of North American ESRD programs reached $25.2 billion in 2002, an 11.5% increase over the previous year, and is expected to reach $29 billion by 2010 [2]. Two factors, aging and the global epidemic of type-II diabetes mellitus, are primarily responsible for the increasing incidence of CKD in adults.

In contrast, pediatric ESRD patients (<20 years of age) constitute a very small proportion of the total ESRD population. However, they pose unique challenges to providers and to the health care system, which must address not only the primary renal disorder but the many extrarenal manifestations that affect growth and development as well. In North America, children younger than 20 years of age account for less than 2% of the total ESRD patient population, and the prevalence of patients aged 0–19 years has grown a modest 32% since 1990. This is in contrast to the 126% growth experienced by the entire ESRD population over the same time period [5]. Nonetheless, CKD in children is a devastating illness, and the mortality rate for children with ESRD receiving dialysis therapy is between 30 and 150 times that of the general pediatric population [6, 7]. In fact, the expected remaining lifetime for a child 0–14 years of age and on dialysis is only 20 years [6]. Therefore, the diagnostic and therapeutic approach to CKD must emphasize primary prevention, early detection, and aggressive management. Knowledge of the epidemiology of CKD and its associated clinical manifestations is a crucial component of this effort by helping to target key patient populations at risk, by quantifying the extent of the problem, and by facilitating an assessment of the impact of intervention.

## Classification of CKD

There is limited information on the epidemiology of CKD in the pediatric population. This is especially true for less advanced stages of renal impairment that are potentially more susceptible to therapeutic interventions aimed at changing the course of the disease and avoiding ESRD. As CKD is often asymptomatic in its early stages, it is both underdiagnosed and, as expected, underreported. This is in part the result of the historical absence of a common definition of CKD and a well-defined classification of its severity. The current CKD classification system described by the National Kidney Foundation’s Kidney Disease Outcomes Quality Initiative (NKF-K/DOQI) has helped remedy the situation. According to the K/DOQI scheme, CKD is characterized by stage 1 (mild disease) through stage 5 (ESRD) (Table 1) [8]. By establishing a common nomenclature, staging has been helpful for patients, general health care providers, and nephrologists when discussing CKD and anticipating comorbidities and treatment plans. The classification system has, however, been subject to debate, as it is argued that stages 1 and 2 would be better defined by the associated abnormalities (e.g. proteinuria, hematuria, structural anomalies) rather being classified as CKD, whereas more advanced stages (3 and 4) should be characterized by the severity of the impaired renal solute clearance [9]. Furthermore, and with particular reference to children, the normal level of glomerular filtration rate (GFR) varies with age, gender, and body size and increases with maturation from infancy, approaching adult mean values at approximately 2 years of age (Table 2). In turn, GFR ranges that define the five CKD stages apply only to children 2 years of age and older. Finally, although the threshold of GFR reduction where chronic renal failure (CRF) and chronic renal insufficiency (CRI) begins is a matter of opinion, many registries have operationally defined this as a GFR below 75 mL/min per 1.73 m^2^ [10]. Hence, populations with CRI or CRF are now categorized as those that comprise CKD stages 2–4.

**Table 1:** National Kidney Foundation’s Kidney Disease Outcomes Quality Initiative (NKF-K/DOQI) stages of chronic kidney disease [8]

Stage	Description	GFR (mL/min/1.73 m^2^)
1	Kidney damage with normal or increased GFR	>90
2	Kidney damage with mild decrease in GFR	60–89
3	Moderate decrease in GFR	30–59
4	Severe decrease in GFR	15–29
5	Kidney failure	<15 or dialysis

*GFR* glomerular filtration rate

**Table 2:** Normal glomerular filtration rate (GFR) in children and adolescents [8]

Age	Mean GFR±SD (mL/min/1.73 m^2^)
1 week (males and females)	41 ± 15
2–8 weeks (males and females)	66 ± 25
>8 weeks (males and females)	96 ± 22
2–12 years (males and females)	133 ± 27
13–21 years (males)	140 ± 30
13–21 years (females)	126 ± 22

## Sources of pediatric data

Most of the existing data on the epidemiology of CKD during childhood concentrates on the late and more severe stages of renal impairment [11, 12] and are not population based in nature [13]. In addition, some methodologically well-designed childhood CKD registries are limited by being restricted to small reference populations [14–16]. Finally, direct comparisons of the incidence and prevalence rate of childhood CKD in different geographical areas around the world is difficult due to methodological differences in study age group, characterization of the degree of renal insufficiency, and disease classification.

In the United States, data is primarily available from two sources: the registry of the North American Pediatric Renal Trials and Collaborative Studies (NAPRTCS) organization [10] and the United States Renal Data System (USRDS). NAPRTCS was established as a transplant registry in 1987 with a goal of gathering data from the majority of pediatric renal transplant centers in the United States, Canada, Mexico, and Costa Rica. Its registry was expanded in 1992 to include data from patients receiving maintenance dialysis, and in 1994, data was first collected from patients with CRI characterized by a Schwartz estimated creatinine clearance of ≤75 mL/min per 1.73 m^2^ [17]. Participation in this registry is voluntary and mandates the involvement of a pediatric nephrologist in the provision of care to those patients entered into the registry. As of December 2005, information had been collected on more than 6,400 patients who entered the registry with a diagnosis of CRI [10].

In contrast to the NAPRTCS, which only receives data voluntarily submitted by pediatric nephrology centers, the USRDS is a national data system that collects, analyzes, and distributes information about all patients with ESRD in the United States. Thus, USRDS data includes information on both adults and children with stage 5 CKD, which is published as an Annual Data Report (ADR) [5, 6]. This source of information is particularly important from an epidemiological perspective, as approximately one third of children and adolescents with ESRD requiring dialysis or transplantation in the United States are cared for in facilities that primarily serve adults, and thus, they are not included in the NAPRTCS database [18].

The recently published data from the ItalKid Project is by far the most comprehensive data on the epidemiology of CKD in children. The ItalKid Project is a prospective, population-based registry that was started in 1990 and includes all incident and prevalent cases of CRF (C_Cr_ < 75 mL/min per 1.73 m^2^) in children (<20 years) from throughout Italy (total population base: 16.8 million children) [19].

The European Dialysis and Transplant Association (EDTA) was established in 1964 to record demographic data and treatment details of patients receiving renal replacement therapy (RRT), including dialysis and renal transplantation. Historically, the EDTA registry gathered data on RRT in children from individual renal units by means of center and patient questionnaires, a process that was subject to underreporting. At the turn of the century, the EDTA office moved to Amsterdam and began collecting data on RRT entirely through national and regional registries and recently reported data on RRT in children from 12 registries located in Europe (*vide infra*) [20].

Other regional societies, such as the Japanese Society for Pediatric Nephrology (JSPN), have also provided useful epidemiological information. In Japan, children are screened annually by urinalysis in a nationwide program, an approach that has provided invaluable epidemiological information and the opportunity for establishing clinical trials focusing on early detection and intervention. Epidemiological data is also available from Australia and New Zealand [21].

In contrast, epidemiological information from Asia, where 57% of the world’s population resides and a geographic region characterized by a very high proportion of children, is very scant and is primarily based on patients referred to tertiary medical centers [22, 23]. The situation in central and southern Africa or in the Arab countries of North Africa and the Middle East is even more unfortunate, as there are no regional pediatric nephrology societies in place to collect and publish any valid epidemiological data.

## Incidence and prevalence of CKD in childhood

Large population-based studies, such as the Third National Health and Nutrition Examination Survey (NHANES III), have made it possible to estimate the incidence and prevalence of CKD in the adult population [1]. According to this report, the prevalence of patients with early stages of CKD (stages 1–4; 10.8%) is approximately 50 times greater than the prevalence of ESRD (stage 5; 0.2%). There is no comparable information available in the United States on the prevalence of the earlier stages of CKD in children and its relationship to ESRD. This is, in large part, due to differences in disease etiology for children and adults. Furthermore, the relationship between the prevalence of earlier stages of CKD and the subsequent development of more severe CKD/ESRD is determined in part by factors unrelated to disease etiology, as was recently shown in a comparison between adult patients in Norway and the United States [4]. Data that do exist on the epidemiology of CKD in children come from a variety of sources.

Population-based data from Italy (ItalKid Project) has reported a mean incidence of preterminal CKD (C_Cr_ < 75 mL/min per 1.73 m^2^) of 12.1 cases per year per million of the age-related population (MARP), with a point prevalence of 74.7 per MARP in children younger than 20 years of age [19]. The national survey performed in Sweden from 1986 until 1994 included children (ages 6 months to 16 years) with more severe preterminal CKD (C_Cr_ < 30 mL/min per 1.73 m^2^) and reported a median annual incidence and prevalence of 7.7 and 21 per MARP, respectively [16]. Similarly, the incidence rate of severe pre-terminal CKD in Lorraine (France) has been estimated as 7.5 per MARP in children younger than 16 years; the prevalence rate ranged from 29.4 to 54 per MARP [15]. In Latin America, the Chilean survey from 1996 reported incidence and prevalence rates of 5.7 and 42.5 per MARP, respectively, in children younger than 18 years of age with C_Cr_ < 30 mL/min/1.73 m^2^, including patients with ESRD [12]. As alluded to above, there are 81.2 million children in the United States younger than 20 years of age [5], but no data on the incidence or prevalence of preterminal CKD is available.

Due to a lack of national registries, any semblance of incidence and prevalence data from developing countries primarily originates as reports from major tertiary care referral centers [22–27]. The nature of the data depends on local referral practices and accessibility to hospital care. The Jordan University Hospital has estimated the annual incidence and prevalence of severe CKD (C_Cr_ < 30 mL/min per 1.73 m^2^) to be 10.7 and 51 per MARP, respectively, based on their hospital admission rate [26]. A 15-year review of admissions from a university teaching hospital in Nigeria estimated the median annual incidence of severe CKD (C_Cr_ < 30 mL/min per 1.73 m^2^) to be 3.0 per MARP, with a prevalence of 15 patients per million children [27]. In a recent report, data from a major tertiary hospital in India revealed that approximately 12% of patients (*n* = 305) seen by the pediatric nephrology service over a 7-year period had moderate to severe CKD (C_Cr_ < 50 mL/min per 1.73 m^2^), and one quarter of these patients had already developed ESRD, highlighting the late diagnosis and referral pattern [23]. Similar data was reported from another tertiary hospital in India where 50% of 48 patients presenting with CRF over a 1-year period had ESRD [22]. Finally, data from a major Iranian hospital collected over 7 years (1991–1998) reported that 11% of pediatric nephrology admissions (*n* = 298) were due to severe CKD (C_Cr_ < 30 mL/min per 1.73 m^2^), and one half of the patients advanced to ESRD [25].

The incidence rate of ESRD, adjusted for race and gender, is much higher among adults than among children. Data from the USRDS revealed that in pediatric patients younger than 20 years of age, the annual incidence of ESRD increased marginally from 13 per MARP in the 1988 cohort to 15 per MARP in the 2003 cohort [5]. This is in contrast to the adult incidence rate of 119 per MARP for patients 20–44 years of age and 518 per MARP for those 45–64 years old in the 2003 cohort [5]. As in adults, a higher incidence rate with older patients was also found across the 5-year age groups within the pediatric cohort. The incidence rate was nearly twice as high among children 15–19 years of age (28 per MARP) compared with children 10–14 years of age (14 per MARP), and nearly three times higher than the rate for children 0–4 years of age (9 per MARP). The point prevalence for pediatric patients (adjusted for age, race, and gender) was 82 per million population during 2002–2003 [5].

The EDTA registry recently reported its cumulative data on 3,184 patients (<20 years of age) with ESRD who initiated RRT between 1980 and 2000 in 12 European countries [20]. With a total of 18.8 million children between 0–19 years in the countries surveyed, data revealed that the incidence of ESRD rose modestly from 7.1 per MARP in the 1980–1984 cohort to 9.9 per MARP over the next 15 years. In contrast, the prevalence of patients receiving RRT increased from 22.9 per MARP in 1980 to 62.1 per MARP in 2000, providing evidence of improved long-term survival. As in the United States, the incidence of ESRD was highest in the 15–19 year age group, with the exception of the 0- to 4-year age group in Finland who experienced a high incidence of ESRD (15.5 per MARP) secondary to the large number of infants in that country with congenital nephrotic syndrome. The incidence of ESRD in children (<20 years age) from Australia and New Zealand has remained fairly constant at around 8–10 per million population over the past 25 years, whereas the prevalence of treated ESRD has steadily increased since 1980, from approximately 25 to 50 patients per million population [21].

The 1998 Japanese National Registry data reported comparatively lower ESRD incidence and prevalence rates of 4 and 22 per MARP, respectively, for children 0–19 years of age [28] for reasons that are as yet unexplained. However, as in other countries, the prevalence rate of treated ESRD patients among patients aged 15–19 years of age was not only high (34 per million), but seven times higher than that of patients 0–4 years of age (5 per million).

**Fig. 1:**
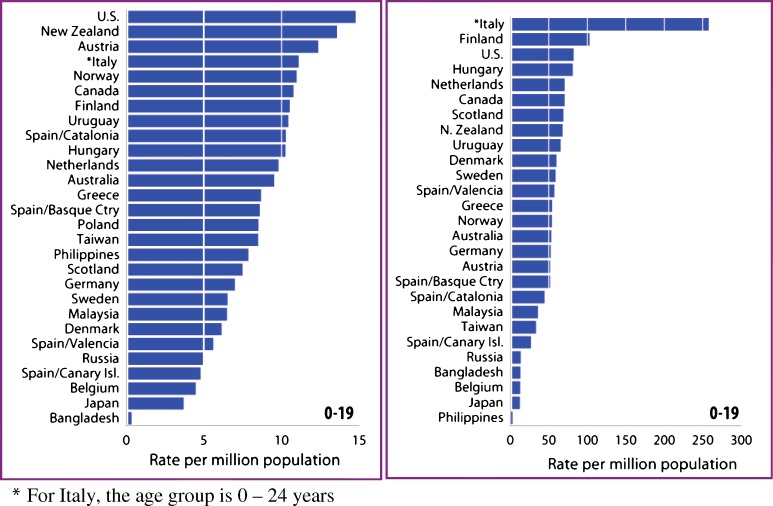
Incidence (*left*) and prevalence of end-stage renal disease (ESRD) around the world in the 0–19 age group in 2003 [5]

In the 2005 ADR from the USRDS, data regarding the incidence and prevalence of ESRD in children was simultaneously published from 37 countries to corroborate the information above and to facilitate international comparisons [5]. The highest incidence rates for children were reported from the United States, New Zealand, and Austria, at 14.8, 13.6, and 12.4 per million population, respectively. As mentioned earlier (*vide supra*), Japan’s rate for pediatric patients was, in contrast, one of the lowest, even though Japan ranks fourth highest in the world for the incidence of ESRD in adults. The prevalence rate for pediatric ESRD patients was reported to be highest in Italy, at 258 patients per million population; however, this may be partially related to the addition of data from patients ages 20–24 to the prevalent group. The second highest prevalence rate for children was reported from Finland, with a rate only 40% of that in Italy but greater than the rates from the United States and Hungary, where they were reported to be 82 and 81 patients per million population, respectively (Fig. 1) [5].

A number of factors influence incidence and prevalence rate variability of childhood ESRD. Factors such as racial and ethnic distribution, type of prevalent renal disease, and quality of medical care available for preterminal CKD patients have a significant impact on patient outcome. As the vast majority of treated ESRD patients come from more-developed countries, which can afford the cost of renal replacement therapy, the huge disparity in the prevalence of ESRD between the more- and less-developed countries probably stems, in large part, from the inadequacy of health-care resource allocation to programs providing renal replacement therapy in underdeveloped countries [29, 30].

Finally, characterization of the patient population with CKD (both preterminal CRF and treated ESRD) reveals that the incidence and prevalence rates are universally greater for boys than for girls [10, 16, 19, 22, 23, 25–27]. Two thirds of patients in the NAPRTCS CRI registry and in the database of the ItalKid Project are males. This gender distribution reflects the higher incidence of congenital disorders, including obstructive uropathy, renal dysplasia, and prune belly syndrome, in boys versus girls. In fact, in the ItalKid Project, males continue to predominate (male:female ratio 1.72) even after excluding patients with posterior urethral valves [19].

As for race, the incidence rate for ESRD in black children in North America is two to three times higher than for white children, irrespective of gender [31]. Likewise, the incidence rate of ESRD for the indigenous people of Australia (Aborigines) and New Zealand (Maoris) is disproportionately higher than that experienced by the remainder of the population [32].

## Etiology of CKD

Unlike adults in whom diabetes and hypertension are responsible for the majority of CKD, congenital causes are responsible for the greatest percentage of all cases of CKD seen in children. However, whereas this is the most common reported etiology from developed countries where CKD is diagnosed in its earlier stages, infectious or acquired causes predominate in developing countries, where patients are referred in the later stages of CKD. These generalizations apart, certain disorders giving rise to CKD are, indeed, more common in some countries than in others.

In the CRI registry arm of NAPRTCS, almost one half of the cases are accounted for by patients with the diagnoses of obstructive uropathy (22%), aplasia/hypoplasia/dysplasia (18%), and reflux nephropathy (8%) (Table 3). Whereas structural causes predominate in the younger patients, the incidence of glomerulonephritis (GN) increases in those older than 12 years. Among the individual glomerular causes, only focal segmental glomerulosclerosis (FSGS) accounts for a significant percentage of patients (8.7%), whereas all other glomerulonephritides combined contribute less than 10% of the causes of childhood CKD. For reasons that are as yet not clear, FSGS is three times more common in blacks than in whites (18% vs. 6%) and is particularly common among black adolescents with CKD [10].

**Table 3:** Diagnosis distribution of North American Pediatric Renal Trials and Collaborative Studies (NAPRTCS) chronic renal insufficiency (CRI) patients [10]

Distributions by diagnosis	Number	Percent Male	Percent white	Percent black	Percent other
Total	6,405	64	61	19	20
Primary diagnosis					
Obstructive uropathy	1,385	86	61	21	17
Aplastic/hypoplastic/dysplastic kidney	1,125	62	62	17	21
Other	913	58	63	16	21
FSGS	557	57	40	39	21
Reflux nephropathy	536	53	74	6	20
Polycystic disease	257	55	74	11	15
Prune belly	185	97	62	23	15
Renal infarct	155	53	66	13	21
Unknown	168	52	47	20	32
HUS	134	58	81	7	11
SLE nephritis	96	25	27	41	32
Cystinosis	97	48	92	3	5
Familial nephritis	99	86	61	12	27
Pyelo/interstitial nephritis	87	39	64	20	16
Medullary cystic disease	82	50	84	9	7
Chronic GN	76	50	43	29	28
MPGN-type I	67	61	48	19	33
Berger’s (IgA) nephritis	64	63	64	16	20
Congenital nephrotic syndrome	68	46	46	12	43
Idiopathic crescentic GN	46	48	52	24	24
Henoch-Schönlein nephritis	40	65	78	3	20
MPGN-type II	29	72	79	3	17
Membranous nephropathy	33	48	30	39	30
Other systemic immunologic disease	25	32	40	32	28
Wilms tumor	28	54	57	21	21
Wegener’s granulomatosis	17	76	94	0	6
Sickle cell nephropathy	13	62	0	92	8
Diabetic GN	11	50	36	45	18
Oxalosis	6	67	83	0	17
Drash syndrome	6	100	67	0	33

*FSGS* focal segmental glomerulosclerosis, *HUS* hemolytic uremic syndrome, *SLE* systemic lupus erythematosus, *GN* glomerulonephritis, *MPGN* membranoproliferative GN, *IgA* immunoglobulin A

Data from the ItalKid Project revealed that hypoplasia with or without urological malformations accounts for as many as 57.6% of all cases of CKD in Italy, whereas glomerular diseases account for as few as 6.8% of cases of CKD in children [19]. Interestingly, when the analysis was restricted to the patient population that had reached ESRD, the relative percentage of glomerular diseases increased from 6.8% to 15.2%, whereas that of hypoplasia decreased from 57.6% to 39.5%, underscoring the discrepancy between the rates of progression of these two entities. Observations from this study have also prompted questions regarding the commonly accepted cause–effect relationship between vesicoureteral reflux (VUR) and kidney disease (reflux nephropathy) and support the hypothesis that both hypoplasia and VUR may be related to similar developmental factors causing congenital disorders of the kidney and urinary tract [33].

In the ESRD population reported by the EDTA registry, hypoplasia/dysplasia and hereditary diseases were the most common causes for ESRD in the 0- to 4-year age group, whereas GN and pyelonephritis became progressively more common with increasing age in the majority of reporting countries [20]. The exception is Finland, where congenital nephrosis (Finnish type) remains the most common cause of ESRD in children younger than 15 years of age [34]. Somewhat different is the data reported by the Japanese National Registry, which reflects a very high proportion (34%) of cases secondary to GN [FSGS 60% and immunoglobulin A (IgA) nephropathy 17%] in their pediatric ESRD population [28]. Similarly, the Australia and New Zealand Dialysis and Transplant (ANZDATA) registry reported GN to be the most common cause of ESRD in children and adolescents from Australia and New Zealand (42%) [21].

Comprehensive information on the etiology of ESRD from many less-developed countries is unavailable owing to poor data collection and the absence of renal registries. In addition and in contrast to the experience within developed countries, many of these countries continue to suffer from the burden of infectious diseases such as hepatitis C, malaria, schistosomiasis, and tuberculosis, with resultant infection-related GN. One such example is Nigeria, from which a publication on pediatric CKD reported various glomerulopathies as the cause of renal failure in one half of their patients, a third of whom also had nephrotic syndrome [27]. Human-immunodeficiency-virus (HIV)-associated nephropathy in children is another entity that is underreported, and it is a disorder that is likely to increase along with the increasing incidence of HIV in Africa and Asia. Familial Mediterranean fever leading to amyloidosis has been found to be responsible for up to 10% of cases of CKD in Turkish children (*n* = 459) [24].

Hereditary disorders are more prevalent in countries where consanguinity is common. One third of Jordanian children with CKD have been diagnosed with hereditary renal disorders such as polycystic kidney disease, primary hyperoxaluria, and congenital nephrotic syndrome [26]. Similarly, one fifth of Iranian children with CKD have been reported to have hereditary disorders such as cystinosis, cystic kidney disease, Alport syndrome, and primary hyperoxaluria [25].

## Progression of CKD

Although the stages of CKD are now reasonably well defined, the natural history of the early stages is variable and often unpredictable. However, most available data demonstrates a slower progression toward ESRD in patients with congenital renal disorders compared with patients with glomerular disease. For this reason, and as alluded to previously, the relative proportion of glomerular diseases increases in groups of patients with more advanced stages of CKD. The progression of established CKD is also influenced by a variety of risk factors, some of which (e.g., obesity, hypertension, and proteinuria) may be modifiable [35–37], whereas others, including genetics, race, age, and gender, are not.

Obesity is associated with hypertension, albuminuria, and dyslipidemia, all of which can potentially influence the progression of CKD. The incidence of certain glomerulonephritides, such as FSGS, is higher in obese than in lean individuals [38, 39]. Hypertension together with proteinuria has been shown to be an important risk factor for progression of primary renal disease in children and adults [40, 41], and the renoprotective efficacy of renin angiotensin system (RAS) antagonists, which is in part independent of blood pressure, has been clearly demonstrated in animal models and adults with acquired nephropathies [42–46]. Whereas both angiotensin-converting enzyme (ACE) inhibitors and angiotensin receptor blockers have been shown to reduce proteinuria in children with CKD, the renoprotective efficacy of these medications in children and their potential impact on the epidemiology of CKD still needs to better delineated, as is currently being addressed by the Effect of Strict Blood Pressure Control and ACE Inhibition on the Progression of Chronic Renal Failure in Pediatric Patients (ESCAPE) trial [47, 48].

The clustering of CKD in families is strongly suggestive of a genetic or familial predisposition in some cases [49]. Studies have suggested the presence of links between CKD and various alterations or polymorphisms of candidate genes encoding putative mediators, including the renin–angiotensin system. Additionally, racial factors may play a role in susceptibility to CKD, as there is a strong concordance of renal disease in the families of African Americans with hypertensive ESRD [49]. Not only may there be an increased susceptibility to disease, but there is evidence that the rate of progression of CKD is faster among African American males [50]. Low birth weight in some ethnic communities might be associated with a reduction in the number of nephrons and a subsequent predisposition to hypertension and renal disease in later life [51].

Irrespective of the underlying kidney disease or presence of additional risk factors, it is clear that the risk of progression to ESRD in childhood is inversely proportional to the baseline creatinine clearance [10, 19]. Additionally, regardless of the initial level of renal insufficiency, puberty seems to be a critical stage for patients with renal impairment, as a steep decline in renal function often occurs during puberty and the early postpuberty period [19]. Whereas the specific reasons are yet to be determined, it is speculated that this pattern of progression may be attributable to an adolescent-specific pathophysiological mechanism, possibly related to sex hormones and/or the imbalance between residual nephron mass and the rapidly growing body size. Data collected by NAPRTCS has also revealed that patients whose baseline serum albumin was below 4 g/dl, inorganic phosphorus above 5.5 mg/dl, calcium below 9.5 mg/dl, blood urea nitrogen (BUN) above 20 mg/dl, or hematocrit below 33% had a significantly higher risk of reaching ESRD (*p* < 0.001) [10]. Data pertaining to a variety of risk factors potentially associated with the progression of CKD, including those noted above, is being collected by the Chronic Kidney Disease in Children Study (CKiD), a prospective, multicenter initiative funded by the National Institutes of Health designed to follow the course of 540 children with CKD for 2–4 years [52].

## Outcome for children with CKD

The outcome of children with severe CKD is highly dependent upon the economy and availability of health care resources. Approximately 90% of treated ESRD patients come from developed countries that can afford the cost of RRT [29]. Despite comparable incidence rates, high mortality in countries that lack resources for RRT results in a low prevalence of CKD patients in those countries. In one of the tertiary care hospitals in India, for example, up to 40% of the ESRD patients opted out of further therapy because of a lack of financial resources [22], and of the 91 patients with ESRD in another hospital, only 15 underwent renal transplantation, 63 received hemodialysis, and the remainder opted out of dialysis or transplantation care secondary to financial constraints [23]. Similar results were recently published from South Africa where only 62% of children (<20 years of age) with ESRD were accepted by an “Assessment Committee” for RRT as part of a rationing program [30].

**Fig. 2:**
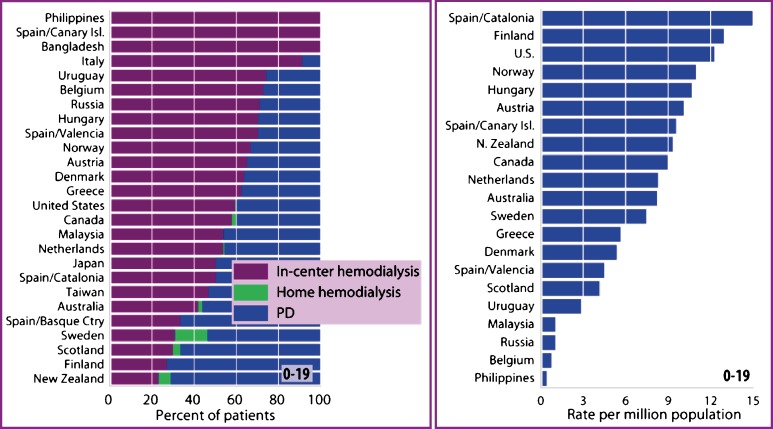
Percent distribution of prevalent dialysis modality (*left*) and transplant rates in the 0–19 age group in 2003 [5]

In countries where RRT is readily available, the most favored renal replacement modality is transplantation in all pediatric age groups. Sixteen percent of children newly diagnosed with ESRD in North America receive a preemptive transplant, and three fourth of children receive a transplant within 3 years of RRT initiation [5]. Similar figures are reported by the ANZDATA registry [21]. Among Western countries, Spain/Catalonia has the highest pediatric transplant rate, reaching 15 patients per million population, followed by a rate of 12 patients per million population in the United States and Finland (Fig. 2) [5]. In the United States, white pediatric patients are more likely to receive a renal transplant than are patients from other racial groups.

The distribution of dialysis modalities varies among countries (Fig. 2) [5]. Peritoneal dialysis (PD) in children is highest in Finland, New Zealand, and Scotland, accounting for 73%, 71%, and 67% of prevalent dialysis patients, respectively. Whereas PD is still the favored mode of dialysis in young children, there has been an increase in hemodialysis (HD) utilization since the early 1990s, and HD is now the most common form of dialysis overall for prevalent patients <19 years of age (Fig. 2) [5]. In the United States, PD is the most frequently used dialysis modality (60% of dialysis patients) according to the NAPRTCS registry [10], whereas HD is more common according to data collected by the USRDS [5]. Once again, this discrepancy reflects in part the fact that many adolescent patients are cared for in adult dialysis units where there is often a preference for HD [18]. Whereas automated PD (APD) is the most frequently used PD modality in children [53], continuous ambulatory PD (CAPD) is commonly used in countries that lack finances and technical support, as reflected in the recent report of the Turkish Pediatric Peritoneal Dialysis (TUPEPD) registry. [54].

Mortality rates remain significantly lower in pediatric patients with ESRD compared with their adult counterparts. Nevertheless, an assessment of the causes of death reflect the excess risk of cardiac and vascular disease and the high prevalence of left ventricular hypertrophy and dyslipidemia among children treated with RRT [55–57]. Pediatric patients with glomerulonephritis or those with cystic/hereditary/congenital disease have the greatest probability of surviving 5 years, in contrast to patients who have developed ESRD as a result of secondary GN or vasculitis [5]. Infants on dialysis have a higher mortality rate than do older children, which is likely, at least in part, to be a result of coexisting morbidities [58]. Although substantial improvement has occurred in the long-term survival of children and adolescents with ESRD over the past 40 years, the overall (dialysis and transplantation) 10-year survival remains at only 80%, and the age-specific mortality rate is still 30–150 times higher than among children without ESRD [6, 7]. It is noteworthy that dialysis is associated with an appreciably higher risk of death compared with renal transplantation; therefore, patients who experience a longer wait for transplantation are more likely to have a worse overall outcome. Not only is the benefit of transplantation evident when one compares transplant recipients to patients deemed “medically unsuitable” for transplantation, it has also been substantiated in a recent longitudinal study of 5,961 patients ≤18 years of age, all of whom were placed on the kidney transplant waiting list in the United States [59]. In that study, transplanted children had a lower estimated mortality rate (13.1 deaths/1,000 patient years) compared with patients on the waiting list (17.6 deaths/1,000 patient years). Similarly, the 2005 ADR reported that approximately 92% of children initiating therapy with a transplant survive 5 years compared with 81% of those receiving HD or PD [5]. Finally, the expected remaining lifetime for children 0–14 years of age and on dialysis is only 18.3 years, whereas the prevalent transplant population of the same age has an expected remaining lifetime of 50 years [5].

## Conclusion

Children with CKD comprise a very small but important portion of the total CKD population. Whereas disorders associated with its development are well delineated, the availability of valid and widespread information regarding the epidemiology of CKD in children requires additional efforts, such as the ItalKid Project, in which early identification and longitudinal follow-up are key practices. This information will, in turn, serve as the basis upon which to judge the impact that observational trials such as CKiD and interventional trials such as ESCAPE have on the evolution of CKD during childhood [48].

## Appendix

Questions: (Answers appear following the reference list)

1. In North America, what percentage of the total ESRD population is constituted by children?
  - 10%
  - 5%
  - Less than 2%
  - 17%
2. What is the most common cause of CKD in children?
  - Glomerulonephritides
  - Focal segmental glomerulosclerosis
  - Chronic pyelonephritis
  - Congenital obstructive uropathy and aplasia/hypoplasia/dysplasia
3. Which of the following disease categories increase in relative frequency with the progression of CKD to ESRD in children?
  - Reflux nephropathy
  - Renal hypoplasia
  - Glomerulonephritis
  - Obstructive uropathy
4. The differences between some of the epidemiological information derived from the NAPRTCS and the USRDS are mainly due to:
  - Data reporting to NAPRTCS is voluntary
  - USRDS collects information on all cases of ESRD and CKD
  - Up to one third of the pediatric patients are cared for in adult dialysis units
  - USRDS obtains information from insurance companies
5. Which developed country has the lowest incidence of pediatric ESRD?
  - United States of America
  - Japan
  - Italy
  - Sweden
6. Which of the following factors is least likely to be associated with the progression of CKD in children?
  - Obesity
  - Puberty
  - Race
  - Gender
7. Which country has the highest pediatric renal transplant rate?
  - United States
  - Finland
  - Germany
  - Spain
8. In the United States, FSGS is most common in:
  - Caucasians
  - Asian immigrants
  - Hispanics
  - Native Americans
  - African Americans
9. Across the 5-year age groups within the pediatric cohort, the highest incidence of ESRD is seen in which age group?
  - 0–4 years
  - 5–9 years
  - 10–14 years
  - 15–19 years
10. Which country has the highest incidence of ESRD in the 0- to 4-year age group?
  - Iran
  - Spain
  - Finland
  - Jordan

Answers:

1. c
2. d
3. c
4. c
5. b
6. d
7. d
8. e
9. d
10. c
